# A *COL7A1* Mutation Causes Dystrophic Epidermolysis Bullosa in Rotes Höhenvieh Cattle

**DOI:** 10.1371/journal.pone.0038823

**Published:** 2012-06-08

**Authors:** Annie Menoud, Monika Welle, Jens Tetens, Peter Lichtner, Cord Drögemüller

**Affiliations:** 1 Institute of Genetics, Vetsuisse Faculty, University of Bern, Bern, Switzerland; 2 Institute of Animal Pathology, Vetsuisse Faculty, University of Bern, Bern, Switzerland; 3 Institute for Animal Breeding and Husbandry, Christian-Albrechts-University Kiel, Kiel, Germany; 4 Institute of Human Genetics, Helmholtz Zentrum München – German Research Center for Environmental Health, Neuherberg, Germany; 5 DermFocus, Vetsuisse Faculty, University of Bern, Bern, Switzerland; Lund University Hospital, Sweden

## Abstract

We identified a congenital mechanobullous skin disorder in six calves on a single farm of an endangered German cattle breed in 2010. The condition presented as a large loss of skin distal to the fetlocks and at the mucosa of the muzzle. All affected calves were euthanized on humane grounds due to the severity, extent and progression of the skin and oral lesions. Examination of skin samples under light microscopy revealed detachment of the epidermis from the dermis at the level of the dermo epidermal junction, leading to the diagnosis of a subepidermal bullous dermatosis such as epidermolysis bullosa. The pedigree was consistent with monogenic autosomal recessive inheritance. We localized the causative mutation to an 18 Mb interval on chromosome 22 by homozygosity mapping. The *COL7A1* gene encoding collagen type VII alpha 1 is located within this interval and *COL7A1* mutations have been shown to cause inherited dystrophic epidermolysis bullosa (DEB) in humans. A SNP in the bovine *COL7A1* exon 49 (c.4756C>T) was perfectly associated with the observed disease. The homozygous mutant T/T genotype was exclusively present in affected calves and their parents were heterozygous C/T confirming the assumed recessive mode of inheritance. All known cases and genotyped carriers were related to a single cow, which is supposed to be the founder animal. The mutant T allele was absent in 63 animals from 24 cattle breeds. The identified mutation causes a premature stop codon which leads to a truncated protein representing a complete loss of COL7A1 function (p.R1586*). We thus have identified a candidate causative mutation for this genetic disease using only three cases to unravel its molecular basis. Selection against this mutation can now be used to eliminate the mutant allele from the Rotes Höhenvieh breed.

## Introduction

Epidermolysis bullosa (EB) is a family of heritable mechanobullous disorders affecting the integrity of the skin and mucosa [1]. Abnormalities of macromolecules, which anchor the dermis to the epidermis lead to diminished cohesion of the skin layers, blister formation, and fragility. The condition is triggered by frictional movement as well as minor trauma. The severity of skin manifestations can be highly variable and is dependent on the mode of inheritance and the underlying mutation [1]. Mutations in 14 genes have been identified as causes of human EB [1], [2]. EB affects the basement membrane zone of the skin and has traditionally been divided into three broad categories based on the cleavage levels of skin: (a) the simplex forms (EBS) demonstrate tissue separation within the basal keratinocytes; (b) the junctional forms (JEB) depict tissue separation within the lamina lucida of the dermal – epidermal basement membrane, and (c) the dystrophic forms (DEB) show tissue cleavage below the lamina densa within the upper papillary dermis [1], [3]. Inherited forms of EB are well known in humans and in several domestic animal species. Various forms of EB have been described in sheep [4], [5], cattle [6], [7], [8], [9], horse [10], dog [11], [12], [13] and cat [14]. In only five EB cases in domestic animals the responsible gene mutation is known [5], [7], [10], [12], [13]. A dominant inherited EBS in Friesian x Jersey crossbred cattle was associated with a *KRT5* mutation [7]. Recessive inherited JEBs were reported in Belgian draft horses (*LAMC2* nonsense mutation [10]), in German pointer dogs (*LAMA3* missense mutation [12]) and in Black headed mutton sheep (*LAMC2* nonsense mutation [5]). Recessive inherited DEB in Golden retriever dogs is caused by *COL7A1* mutations [13] and in Swiss white alpine sheep an inherited *COL7A1* defect is probably causative but the mutation has not been described [4].

Currently, there is no drug therapy available to treat the EB disease. Therefore, studying suitable genetically defined animal models may be useful for the identification of therapeutic targets and approaches [2], [12], [15].

In 2010, an outbreak of EB was noticed in a local German cattle breed. We assessed the clinical presentation of the disease and carried out histopathological examination to define the disease as subepidermal bullous dermatosis. Considering the age of the affected animals dystrophic epidermolysis bullosa (DEB) was suspected. Subsequently, we employed a positional cloning approach to identify the causative mutation in the bovine *COL7A1* gene.

## Results

### Clinical presentation

**Figure 1:**
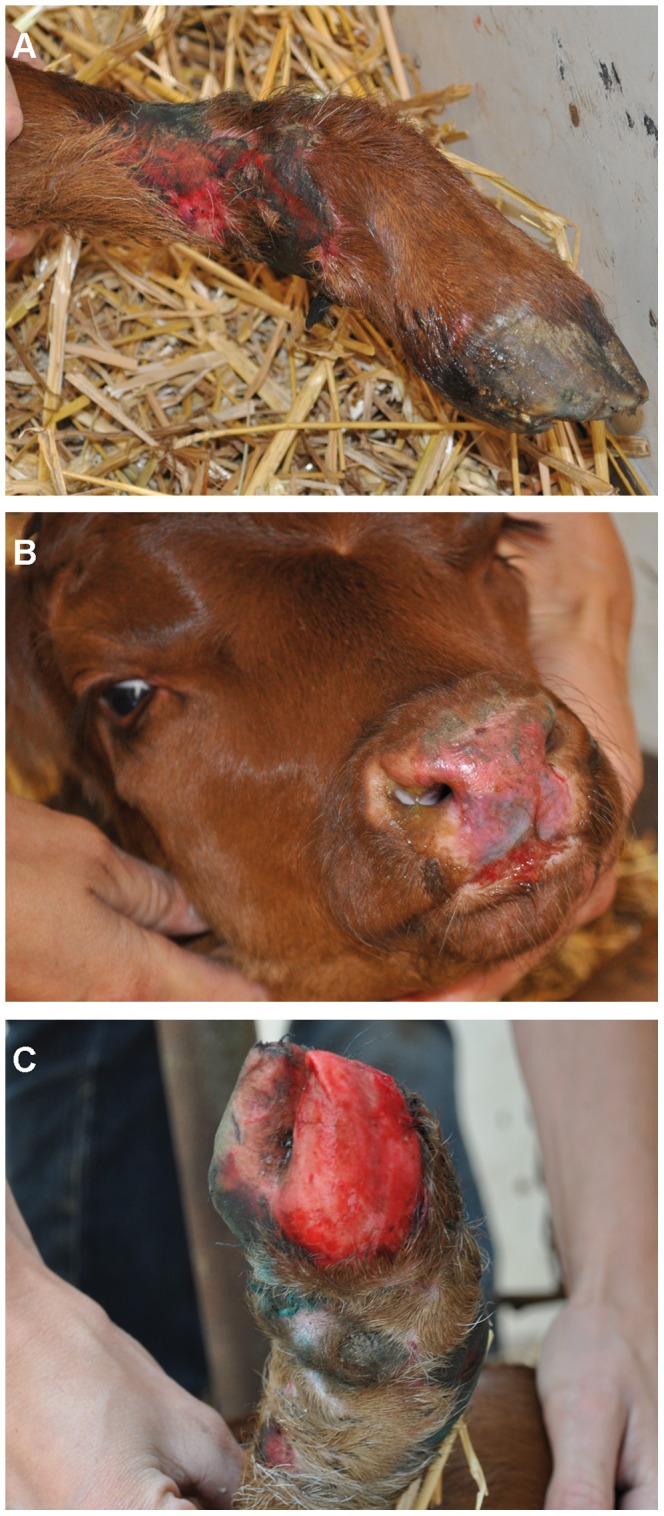
Clinical features of a DEB affected Rotes Höhenvieh calf. Typical signs are the extensive epidermal loss with ulcerations at the fetlocks (A) and muzzle (B) and dysungulation (C).

We ascertained six affected calves in total. The clinical findings from a detailed examined affected bull calf were as follows: the calf was bright immediately post-partum. It was able to stand up, but once standing it was reluctant to walk and therefore could suck his dam only with assistance. It showed extensive epidermal loss at the four fetlocks (Figure 1A). As a consequence of frictional movements the skin defects rapidly extended subsequently involving the entire distal limbs, the whole muzzle and the oral cavity (Figure 1B). The calf showed appetite but was unable to suck, because of the pain it caused. It also showed skin defects around the eyes and at the base of the ears. The skin without epidermis was reddened, edematous, and hairless. The calf developed dysungulation affecting two claws during the first hours of life (Figure 1C). There were no apparent neurological defects or muscle abnormalities. After 8 hours, the calf was recumbent and couldn't stand up anymore because of pain. The calf was euthanized on humane grounds due to the severity, extent and progression of the lesions.

Clinical findings in the other five affected cases were similar and all calves had skin defects at birth at the four fetlocks and skin lesions rapidly extended to the muzzle, the oral cavity and the claws. They all showed extreme fragility of the normal appearing skin. Additional features have been observed in individual cases reported by the owner. In addition, two calves showed ear deformities (one with a closed ear), two other affected calves showed missing dewclaws and one calf had also skin lesions at the tail.

### Histopathology

**Figure 2:**
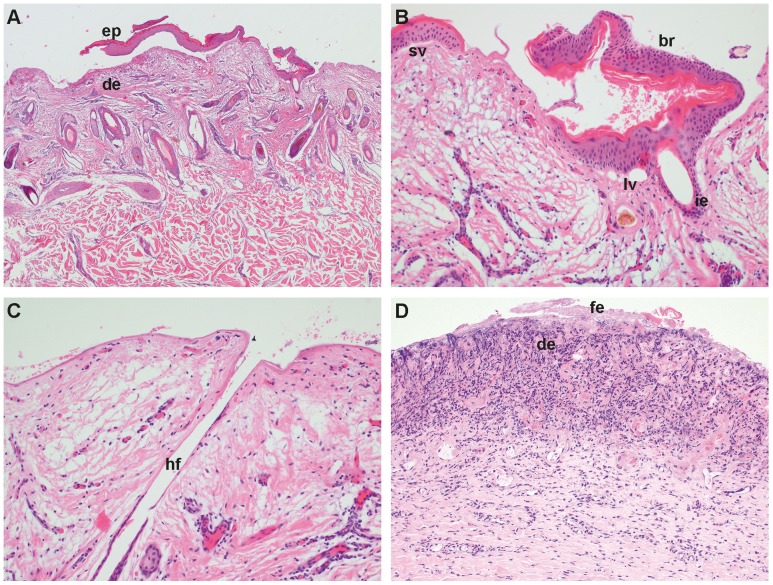
Histopathological features of DEB. Biopsy of the ear of case 1: (A) Note that on the lateral borders of the biopsy the epidermis (ep) is missing and, where present, the epidermis is detached from the underlying dermis (de). The epidermis is cleanly separated from the dermis and the basal cells are intact. H&E, 25×. (B) Higher magnification: Note the intact basal cells of the blister roof (br), larger vacuoles (lv) and a small vesicle (sv) along the basement membrane zone and separation of the infundibular epithelium (ie) from the surrounding connective tissue. H&E, 200× (C) Note that the epidermal and infundibular epithelium of the hair follicle (hf) is missing and the surface is covered by homogenous proteinaceous material. H&E, 200×. Biopsy of the right hind leg of case 3: (D) Necrotic superficial dermis (de) characterized by fibrinous exsudation (fe), cellular debris, dense amounts of degenerate inflammatory cells, and hemorrhage. H&E, 100×.

Histopathological examination revealed in all but one biopsies, a complete absence of the epidermis (Figure 2A–D). Only in one from the ear a long stretch of epidermis was completely detached from the dermis and the clean separation from the underlying dermis indicates that the epidermis is part of a blister roof (Figure 2A). In the same biopsy larger vacuoles and small vesicles along the basement membrane zone are visible (Figure 2B). In the biopsies where hair follicles were present the infundibular epithelium was missing in most cases (Figure 2B, 2C). The denuded dermal surface was covered by homogenous eosinophilic material and in some biopsies nuclear debris and cocci were present within this proteinaceous layer. In the biopsies of another affected calf a mild to severe necrosis of the superficial dermis was present. The necrotic dermis was characterized by fibrinous exudation, cellular debris, variable amounts of degenerate inflammatory cells, congested blood vessels, and hemorrhage (Figure 2D).

### Pedigree analysis

**Figure 3:**
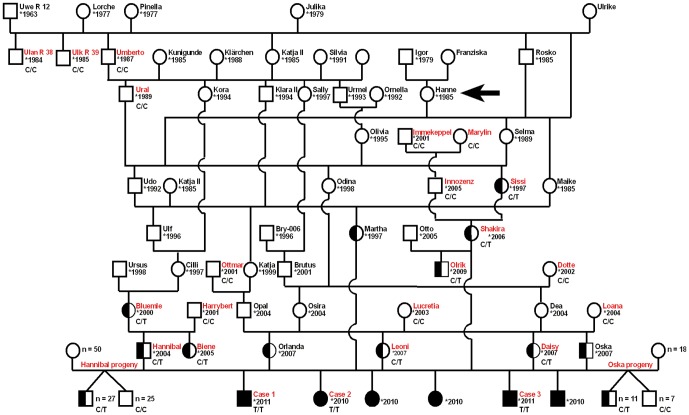
Pedigree of Rotes Höhenvieh cattle with DEB. DNA samples were available only from red labeled cattle. The genotypes for the *COL7A1* c.4756C>T exon 49 SNP are given below the symbols. The arrow indicates the cow, which is supposed to be the founder animal. We genotyped all available male offspring of the two carrier bulls Hannibal and Oska and detected 51% and 61% carriers, respectively.

Initially, we collected samples from three DEB affected calves, a single sire, and three dams of Rotes Höhenvieh cattle on a single farm. The owner reported three additional cases showing similar signs, which occurred during the last 12 months (Figure 3). The parents of the affected cattle showed no clinically visible skin anomalies. Analysis of the pedigree data revealed that two distantly related natural service sires (Hannibal, Oska; Figure 3) had affected offspring among their progeny. The pedigree of the affected calves shows many inbreeding loops (Figure 3). Analysis of the pedigree data revealed that all affected individuals trace back, on both the maternal and paternal path, to a single founder cow (Hanne) born in the year 1985 (Figure 3). The disease was recognized in the year 2010, 3 to 6 generations later. The breeding history was consistent with a monogenic autosomal recessive mode of inheritance.

### Genome-wide homozygosity mapping of the DEB mutation

**Figure 4:**
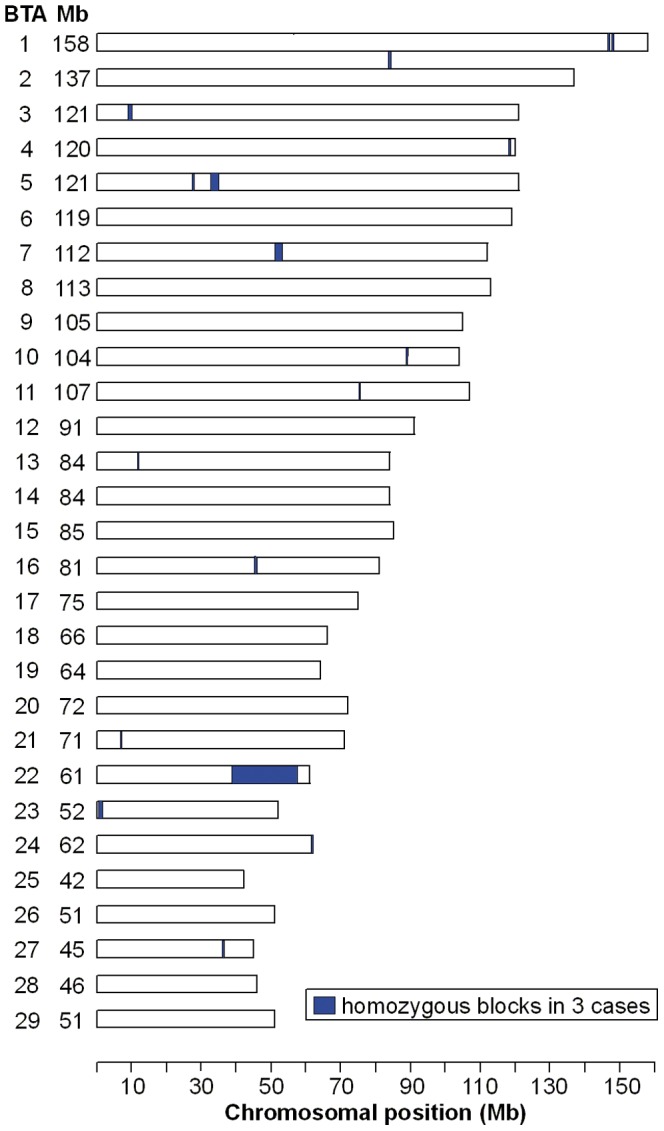
Genome-wide homozygosity mapping of the DEB mutation. After genotyping approximately 777,000 uniformly distributed SNP markers homozygous blocks >0.1 Mb were identified across 3 DEB affected cattle. Only on BTA 22 a very large homozygous block was identified.

Assuming a monogenic recessive inheritance the epidermolysis bullosa affected calves were expected to be identical by descent (IBD) for the causative mutation and flanking chromosomal segments. Therefore, we decided to apply a homozygosity mapping approach to determine the position of the mutation in the bovine genome. We genotyped approximately 777,000 SNPs distributed across the entire genome in 3 cases. We analyzed the 3 genotyped DEB cases for extended regions of homozygosity with simultaneous allele sharing. A total of 17 genomic regions larger than 150 SNP and 0.3 Mb fulfilled our search criteria (Figure 4). The size of homozygous blocks ranged between 0.327 Mb and 18.600 Mb with a mean size of 1.860 Mb and a median of 0.595 Mb. The 3 genotyped cases showed a single large homozygous chromosome region on cattle chromosome (BTA) 22, containing 5,632 SNP markers corresponding to an 18.6 Mb interval from 38.8–57.4 Mb (Figure 4). Analyzing the data of 10 controls from the Rotes Höhenvieh breed genotyped at 54,000 evenly spaced SNPs revealed no shared homozygous regions greater than 300 kb.

### Identification of a functional candidate gene and mutation analysis

As the quality of the bovine genome annotation is not yet perfect, we inferred the gene annotation of the mapped interval from the corresponding human interval. The bovine DEB interval on BTA 22 corresponds to segments on human chromosome (HSA) 3. A careful inspection of HSA 3 genes and database searches of their presumed function revealed *COL7A1* encoding for the collagen type VII alpha 1 as a functional candidate gene within the critical interval at 48.6 Mb on HSA 3 and 51.8 Mb on BTA 22, respectively.

**Figure 5:**
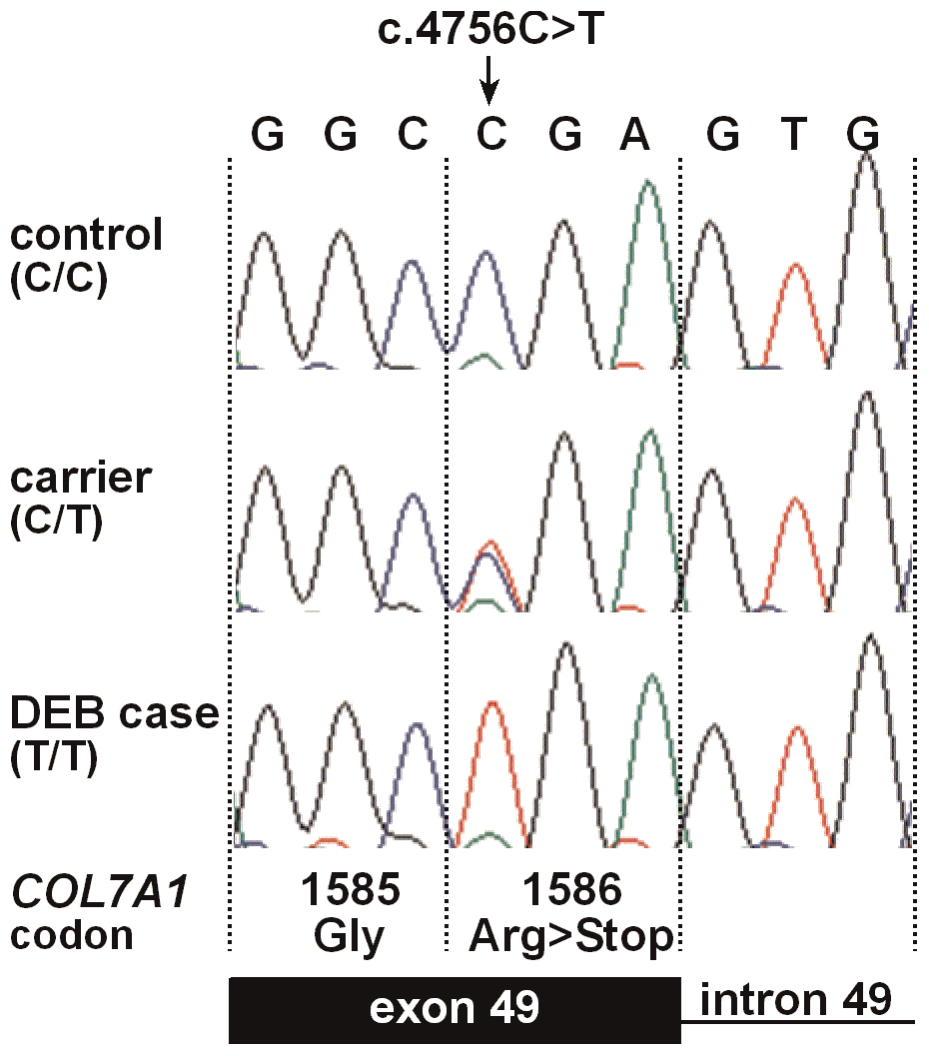
*COL7A1* mutation analysis. Electropherograms of the *COL7A1* c.4756C>T mutation. Representative sequence traces of PCR products amplified from genomic DNA of 3 cattle with the different genotypes are shown. The mutant T allele in homozygous form is present only in DEB affected calves and leads to a premature stop codon.

We performed a mutation analysis in three animals: a single DEB affected calf (case 3, Figure 3), its sire (Hannibal), and its mother (Daisy). We re-sequenced a contiguous genomic interval of more than 30 kb spanning the complete *COL7A1* gene including 118 exons of a total spliced coding length of 8802 bp encoding a protein of 2932 amino acids. Mutation analysis in the three re-sequenced animals revealed 14 single nucleotide polymorphisms (SNPs) in comparison to the cattle reference genome sequence (Table S1). Three SNPs were perfectly associated with the assumed recessive DEB inheritance. Two intronic SNPs were immediately excluded based on the fact that the affected calf was homozygous for the wildtype allele. The remaining SNP is within exon 49 (c.4756C>T; Figure 5). All three available affected calves were homozygous mutant T/T and all 4 supposed carriers were heterozygous C/T for this variant (Table 1). None of 143 healthy Rotes Höhenvieh cattle had the homozygous T/T genotype. All 42 detected C/T carriers of the DEB mutation were directly related to the assumed founder cow (Figure 3). The mutant T-allele was absent from 329 control cattle from 24 diverse cattle breeds (Table 1). The c.4756C>T mutation is predicted to result in a stop codon at amino acid residue 1586 in the bovine COL7A1 protein sequence (p.R1586*).

**Table 1:** *COL7A1* genotype frequencies.

		Rotes Höhenvieh			Other breeds*
c.4756C>T Genotype	DEB affected (n = 3)	DEB carrier (n = 4)	Control, related (n = 86)	Control, unknown relationship (n = 57)	Controls (n = 329)
**CC**			44	57	
**CT**		4	42		
**TT**	3				329

* Angus (n = 18), Aubrac (n = 1), Ayshire (n = 1), Belgian blue (n = 3), Blonde d'Aquitaine (n = 2), Brown Swiss (n = 35), Charolais (n = 17), Chianina (n = 19), Dutch belted (n = 18), Eringer (n = 16), Evolenard (n = 10), Gelbvieh (n = 1), Galloway (n = 20), Hereford (n = 3), Scotish Highland (n = 4), Holstein (n = 35), Jersey (n = 3), Limousin (n = 17), Montbéliarde (n = 4), Nelore (n = 3), Ongola (n = 1), Piedmontese (n = 2), Pinzgauer (n = 10), Pustertaler Sprinzen (n = 10), Romagnola (n = 18), Salers (n = 1), Simmentaler (n = 38), Tyrolean Grey (n = 18), Domestic Yak (n = 1).

## Discussion

In this study we applied a positional cloning approach to unravel the molecular basis of a recently recognized outbreak of dystrophic epidermolysis bullosa (DEB) in Rotes Höhenvieh cattle. Using just a few samples of well diagnosed cases showed again that the availability of genome sequences and high-density SNP genotyping microarrays enables the rapid mapping of causative mutations for monogenic diseases in domestic animal species. The long region of homozygosity surrounding the mutation suggests that the DEB mutation is quite young and that we have correctly identified a single putative founder cow. We note that the mapping of older mutations may require many more cases, because the associated IBD haplotype is usually much smaller, due to independent recombination events over several generations. Thus, we could quickly identify a genomic interval for the DEB mutation in Rotes Höhenvieh cattle containing the *COL7A1* gene, a good functional and positional candidate gene. Based on the recessive mode of inheritance, we expected to find a loss-of-function mutation affecting the coding sequence of *COL7A1*. DNA sequencing revealed a nonsense mutation in the *COL7A1* gene, which is perfectly associated with the DEB phenotype in Rotes Höhenvieh cattle. Possibly nonsense-mediated decay selectively recognizes and degrades mRNAs whose open reading frame is truncated by a premature translation termination codon. We were not able to get fresh skin biopsies for RNA extraction. RT-PCR experiments using extractions from the formalin fixed paraffin embedded material failed. Furthermore, we were not able to detect the COL7A1 protein by immunofluorescence using a human COL7A1 antibody on paraffin sections from skin of affected and normal control animals. Unfortunately, all our samples showed strong background signals of the dermis, therefore we couldn't distinguish specific staining signals from background noise (data not shown). While it is unclear whether the mutant protein of 1585 residues is actually expressed, with more than 46% of the normal COL7A1 protein missing including several collagen triple helix repeats, it is very unlikely that the mutant protein fulfills any physiological function. The dramatically truncated polypeptides likely impair anchoring fibril formation in the homozygous patients. The main component of the anchoring fibrils is collagen VII, a homotrimeric collagen synthesized by keratinocytes and fibroblasts [2], [13], [16]. The type VII procollagen monomer consists of three alpha 1 (VII) polypeptide chains folded into a triple helix. Two monomers form an antiparallel dimer, from which the non-collagen pro-peptides are removed protolytically. Finally, the mature dimers laterally aggregate into anchoring fibrils [17].

Human DEB is caused by mutations in the *COL7A1* gene encoding collagen type VII alpha 1 [1], [16], [18]. Altered expression of collagen type VII results in blisters caused by intradermal separation occurring beneath the lamina densa, at the level of the anchoring fibrils [19], [20]. Loss of collagen VII functions in DEB leads to absence or anomalies of the anchoring fibrils and to dermal-epidermal tissue separation. Numerous recessive inherited severe DEB forms caused by *COL7A1* gene mutations are reported in humans [16], [17], [19], [20], [21]. These severe phenotypes involve extracutaneous lesions in the oral cavity and the gastrointestinal tract. On the other hand, the dominantly inherited *COL7A1* associated DEB phenotypes in humans show milder symptoms [8]. The global severity of the EB diseases in humans can be explained most likely by the fact that the skin is not protected by hair and thus is more susceptible to epidermal detachment as a result of microtrauma. The hair follicles covering most of the body surface in animals may possibly contribute to a better coherence of the skin layers [4], [22]. This may explain the more generalized severe skin lesions in human DEB patients. The clinical appearance of the calves and the histopathologic findings closely correlate with severe generalized recessive dystrophic epidermolysis bullosa in humans.

The observed phenotype resembled cases of epidermolysis bullosa and epitheliogenesis imperfecta reported in different breeds of cattle [23]. Epitheliogenesis imperfecta (EI) is known in veterinary medicine as differential diagnosis for EB but is still not genetically defined. In the past, several cases with similar clinical signs comparable to the recent cases were diagnosed as EI. Because they showed, like the presented DEB cases, only little or no characteristic blistering as seen in milder EB forms, the skin defects were already present at birth and sometimes the individuals showed no ear deformities [23]. Therefore, we suppose that in the past cases of DEB were misdiagnosed as EI.

The Rotes Höhenvieh cattle breed is a traditional German dual purpose cattle breed that was very widespread till the 1950s in Germany. This breed became nearly extinct in the 1980s. A dedicated breeding association (Bundesarbeitgemeinschaft Rotes Höhenvieh) was created to rescue the breed, first to preserve the genetic diversity and, second, because of their particular combination of characteristics like great vitality, longevity, high fertility and efficiency, which are valued traits in animal husbandry. Starting with just a few individuals, the population increased in the 1990s with subsequent breeding of consanguineous individuals. Today there are a total of approximately 100 Rotes Höhenvieh bulls and 1,000 cows. Due to the intense use of very few single artificial insemination (AI) sires the inbreeding rate of the current population is quite high [24]. The pedigree of the presented DEB cases shows inbreeding (Figure 3). Therefore, this study represents an example that this breeding practice can lead to high frequencies of deleterious recessive alleles within a few generations [25]. Homozygosity mapping identified a quite large IBD segment on BTA 22. The long region surrounding the mutation suggests that the identified *COL7A1* mutation is quite young. The founding mutation presumably happened in the cow Hanne (Figure 3). All affected calves and all genotyped carriers of the *COL7A1* mutation can be traced back to this cow born in 1985. The popular AI sire Uwe-R12, which had a significant impact during the consolidation phase of this breed in the 1990s is not involved in the dissemination of the DEB mutation.

In conclusion, we have identified a nonsense mutation of the bovine *COL7A1* gene as the likely causative mutation for DEB in Rotes Höhenvieh cattle. The knowledge on *COL7A1* mutations in human DEB patients suggests that this loss of function mutation is also responsible for the inherited DEB in Rotes Höhenvieh cattle, although we have no functional proof. Our study is consistent with the critical function of collagen type VII alpha 1 for formation of anchoring fibrils in the dermal-epidermal junction.

Our finding enables genetic testing and the eradication of this genetic disease from the breeding population. It is essential that domestic animal populations with small effective population sizes are continuously monitored for the appearance of recessive defects, so that selection against deleterious alleles can be implemented as early as possible.

## Materials and Methods

### Ethics Statement

All animal work has been conducted according to the national and international guidelines for animal welfare. There is no permit number as this study is not based on an invasive animal experiment. The cattle owner agreed that the samples can be used for our study. The data were obtained during diagnostic procedures that would have been carried out anyway. This is a very special situation in veterinary medicine. As the data are from client-owned cattle that underwent veterinary exams, there was no “animal experiment” according to the legal definitions in Germany.

### Animals

We collected blood and skin samples from 3 dystrophic epidermolysis bullosa affected calves (2 male, 1 female) from a single farm. We did a full clinical exam of one affected calf. Additional information about the phenotype was obtained from owner records. Two cases were seen by a local veterinarian. Additionally, we collected 4 samples recorded as parents (1 sire, 3 dams) of affected offspring which were classified as obligate carriers and 143 healthy Rotes Höhenvieh cattle resulting in a total of 150 samples from this breed. Furthermore, we sampled 329 healthy control cattle from 24 genetically diverse cattle breeds for the re-sequencing of *COL7A1* exon 49 (Table 1). Genomic DNA was isolated using the Nucleon Bacc2 kit (GE Healthcare).

### Histopathological examination of skin biopsies

Immediately after euthanasia 6 mm punch biopsies were taken from the skin of two affected calves. Biopsies from one DEB affected calf (case 1, Figure 3) were taken from the right and left foreleg, the right hind leg, the ear and the muzzle on day one after birth. Biopsies from another DEB calf (case 3, Figure 3) were taken one day after birth from lesional skin of the right hind and the right foreleg, the muzzle and the medial canthus of the eye. Skin biopsies were fixed in 10% buffered formalin, processed routinely and the resulting 4 µm sections were stained with haematoxylin and eosin (H&E).

### Genome-wide homozygosity mapping of the DEB mutation

Genomic DNA from 3 cases was genotyped using Illumina's BovineHD BeadChip with 777,962 SNPs [26]. Genomic DNA from 10 controls was genotyped using Illumina's BovineSNP50 BeadChip with 54,001 SNPs [27]. The results were analyzed with PLINK [27]. After removing 12,098 SNPs with low genotyping success (failed calls>0.1) the average genotyping rate per individual was 99.9% for the three cases. After removing 6,287 SNPs with low genotyping success (failed calls>0.1) the average genotyping rate per individual was 98.2% for the ten controls. To identify extended homozygous regions with allele sharing across all affected animals the options –homozyg-group and –homozyg-match were applied. All given bovine genome positions correspond to the UMD3.1 cattle assembly.

### Mutation analysis of the bovine *COL7A1* gene

Genomic DNA of a trio (a single case and both parents, which were assumed to be heterozygous carriers of the mutation) was used for mutation analysis (primer sequences available on request). We amplified PCR products using QIAGEN Multiplex PCR Kit (Qiagen). PCR products were directly sequenced on an ABI 3730 capillary sequencer (Applied Biosystems) after treatment with exonuclease I and shrimp alkaline phosphatase. We analyzed sequence data with Sequencher 4.9 (GeneCodes). All given positions correspond to the bovine *COL7A1* mRNA reference sequence XM_002697051. For the detection of the *COL7A1* exon 49 (c.4756C>T) mutation we amplified a PCR product of 605 bp using a forward primer located in intron 48 (5′-GGCTGATCGTCTTTGTCACC-3′) and reverse primer located in intron 49 (5′-TCAGTCCTGATCCCCAACTC-3′) which was subsequently sequenced as described above.

## Supporting Information

Table S1 Polymorphisms and genotypes of 3 Rotes Höhenvieh cattle in the region of the *COL7A1* gene.
(XLS)

## References

[1] Fine JD, Robin AJ, Bauer EA, Bauer JW, Bruckner-Tuderman L (2008). The classification of inherited epidermolysis bullosa (EB): report of the third international consensus meeting on diagnosis and classification of EB.. J Am Acad Dermatol.

[2] Fine JD (2010). Inherited epidermolysis bullosa: recent basic and clinical advances.. Curr Opin Pediatr.

[3] Fine JD, Eady RA, Bauer EA, Briggaman RA, Bruckner-Tuderman L (2000). Revised classification system for inherited epidermolysis bullosa: report of the second international consensus meeting on diagnosis and classification of epidermolysis bullosa.. J Am Acad Dermatol.

[4] Bruckner-Tuderman L, Guscetti F, Ehrensperger F (1991). Animal model for dermolytic mechanobullous disease: sheep with recessive dystrophic epidermolysis bullosa lack collagen VII.. J Invest Dermatol.

[5] Mömke S, Kerkmann A, Wöhlke A, Ostmeier M, Hewicker-Trautwein M (2011). A frameshift mutation within LAMC2 is responsible for Herlitz type junctional epidermolysis bullosa (HJEB) in black headed mutton sheep.. PloS ONE.

[6] Foster AP, Skuse AM, Higgins RJ, Barrettx DC, Philbeyx AW (2010). Epidermolysis bullosa in calves in the United Kingdom.. Science Direct.

[7] Ford CA, Stanfield AM, Spelman RJ, Smits B, Ankersmidt-Udy AE (2005). A mutation in bovine keratin 5 causing epidermolysis bullosa simplex, transmitted by a mosaic sire.. J Invest Dermatol.

[8] Thompson KG, Crandell RA, Rugeley WW, Sutherland RJ (1985). A mechanobullous disease with sub-basilar separation in Brangus calves.. Vet Pathol.

[9] Medeiros GX, Riet-Correa F, Armién AG, Dantas AF, de Galiza GJ (2012). Junctional epidermolysis bullosa in a calf.. J Vet Diagn Invest.

[10] Spirito F, Charlesworth A, Linder K, Ortonne JP, Baird J (2002). Animal models for skin blistering conditions: absence of laminin 5 causes hereditary junctional mechanobullous disease in the belgian horse.. J Invest Dermatol.

[11] Palazzi X, Marchal T, Chabanne L, Spadafora A, Magnol JP (2000). Inherited dystrophic epidermolysis bullosa in inbred dogs: a spontaneous animal model for somatic gene therapy.. J Invest Dermatol.

[12] Guaguere E, Capt A, Spirito F, Meneguzzi G (2003). Junctional epidermolysis bullosa in the german shorthaired pointer: a spontaneous model for junctional epidermolysis bullosa in man.. Bull Acad Vet France.

[13] Magnol JP, Pin D, Palazzi X, Lacour JP, Gache Y (2005). Characterization of a canine model of dystrophic bullous epidermolysis (DBE). Development of a gene therapy protocol.. Bull Acad Natle Med.

[14] Olivry T, Dunston SM, Marinkovich MP (1999). Reduced anchoring fibril formation and collagen VII immunoreactivity in feline dystrophic epidermolysis bullosa.. Vet Pathol.

[15] Baldeschi C, Gache Y, Rattenholl A, Bouillé P, Danos O (2003). Genetic correction of canine dystrophic epidermolysis bullosa mediated by retroviral vectors.. Human Molecular Genetics.

[16] Christiano AM, Greenspan DS, Hoffman G, Zhang X, Tamai Y (1993). A missense mutation in type VII collagen in two affected siblings with recessive dystrophic epidermolysis bullosa.. Nat Genet.

[17] Kern JS, Kohlhase J, Bruckner-Tuderman L, Has C (2006). Expanding the COL7A1 mutation database: novel and recurrent mutations and unusual genotype-phenotype constellations in 41 patients with dystrophic epidermolysis bullosa.. J Invest Dermatol.

[18] Uitto J (2004). Epidermolysis bullosa: the expanding mutation database.. Soc Inv Derm 123, xii–xiii.

[19] Christiano AM, Suga Y, Greenspan DS, Ogawa H, Uitto J (1995). Premature termination codons on both alleles of the type VII collagen gene (COL7A1) in three brothers with recessive dystrophic epidermolysis bullosa.. J Clin Invest.

[20] Hovnanian A, Hilal L, Blanchet-Bardon C, De Prost Y, Christiano AM (1994). Recurrent nonsense mutations within the type VII collagen gene in patients with severe recessive dystrophic epidermolysis bullosa.. Am J Hum Genet.

[21] Kern JS, Grüninger G, Imsak R, Müller ML, Schumann H (2009). Forty-two novel COL7A1 mutations and the role of a frequent single nucleotide polymorphism in the MMP1 promoter in modulation of disease severity in a large european dystrophic epidermolysis bullosa cohort.. Br J Dermatol.

[22] Fritsch A, Loeckermann S, Kern JS, Braun A, Bösl MR (2008). A hypomorphic mouse model of dystrophic epidermolysis bullosa reveals mechanisms of disease and response to fibroblast therapy.. J Clin Invest.

[23] Bähr C, Drögemüller C, Distl O (2004). Epitheliogenesis imperfecta bei deutschen Holsteinkälbern (German).. Tierärztl Prax 32 (G).

[24] Kehr C, Klunker M, Fischer R, Groeneveld E, Bergfeld U (2010). Untersuchungen zu einem Monitoring genetischer Diversität bei Nutztierrassen: Ergebnisse zum Roten Höhenvieh (German).. Züchtungskunde.

[25] Drögemüller C, Reichart U, Seuberlich T, Oevermann A, Baumgartner M (2011). An unusual splice defect in the mitofusin 2 gene (MFN2) is associated with degenerative axonopathy in Tyrolean Grey cattle.. PLoS ONE.

[26] Homepage Illumina (2011). Accessed 28 December 2011. http://www.illumina.com.

[27] Homepage PLINK (2011). Accessed 28 December 2011. http://pngu.mgh.harvard.edu/~purcell/plink/.

